# Rumen Microbiota of Tibetan Sheep (*Ovis aries*) Adaptation to Extremely Cold Season on the Qinghai-Tibetan Plateau

**DOI:** 10.3389/fvets.2021.673822

**Published:** 2021-05-25

**Authors:** Qingshan Fan, Xiongxiong Cui, Zhaofeng Wang, Shenghua Chang, Metha Wanapat, Tianhai Yan, Fujiang Hou

**Affiliations:** ^1^State Key Laboratory of Grassland Agro-Ecosystems, Key Laboratory of Grassland Livestock Industry Innovation, Ministry of Agriculture, College of Pastoral Agriculture Science and Technology, Lanzhou University, Lanzhou, China; ^2^Department of Animal Science, Faculty of Agriculture, Tropical Feed Resources Research and Development Center (TROFREC), Khon Kaen University, Khon Kaen, Thailand; ^3^Agri-Food and Biosciences Institute, Hillsborough, United Kingdom

**Keywords:** Tibetan sheep, growth performance, digestibility, Firmicutes/Bacteroidetes, VFA profiles

## Abstract

The Qinghai-Tibet Plateau is characterized by low temperatures and hypoxia, and this feature is more obvious in the winter. However, it is not clear how Tibetan sheep adapt to extreme cold climates. To address this, we used physiological methods combined with next-generation sequencing technology to explore the differences in growth performance, forage nutrient digestion, serum biochemical indexes, and rumen microbial communities of Tibetan sheep (*Ovis aries*) between the summer and winter. In the summer, owing to the high nutritional quality of the forage, the Tibetan sheep showed enhanced forage degradation and fermentation though increased counts of important bacteria in the rumen, such as Bacteroidetes, *Prevotella_1, Prevotellaceae_UCG-003, Ruminococcus_1, Saccharofermentans*, and *Ruminococcaceae_UCG-014*, to improve the growth performance and increase serum immunity and antioxidant status. In the winter, owing to the low nutritional quality of the forage, the Tibetan sheep presented low values of forage degradation and fermentation indicators. The relative abundance of Firmicutes, the Firmicutes/Bacteroidetes ratio, microbial diversity, interactive activity between microorganisms, and metabolism were significantly increased, implying that the rumen microbiota could promote the decomposition of forage biomass and the maintenance of energy when forage nutritional value was insufficient in the winter. Our study helps in elucidating the mechanism by which Tibetan sheep adapt to the high-altitude harsh environments, from the perspective of the rumen microbiota.

## Introduction

Interactions between plants and animals are one of the key forces promoting ecosystem evolution and maintaining the structure and function of ecosystems (1). Grasslands are the largest of the terrestrial ecosystems, occupying over half of the earth's land surface. Ruminant livestock depend on grasslands to produce food, milk, wool, and leather worldwide (2). As one of the most critical conflicts in grassland management, seasonal disequilibrium between forage supply, and livestock nutrient demand of livestock inevitably occurs annually; the uneven seasonal distribution of annual precipitation or heat results in excessive forage supply in the wet/warm season and insufficient forage production in the dry/cold season, whereas the nutritional demand of livestock remains relatively steady or only slightly fluctuates throughout the year (3). Unreasonable solutions have caused overgrazing and immediate grassland degradation, which have markedly elevated the risk of ecological disequilibrium and reduction of animal food production (4). Previous studies have mainly focused on the equilibrium of forage mass and quality between grassland supply and livestock demand generally in terms of grassland productivity at the farm scale; however, it is necessary to understand the mechanism of forage mass and quality disequilibrium between grassland supply and livestock demand, especially with respect to rumen microbes and nutrient physiology of livestock.

The rumen is an important digestive compartment that contains diverse microorganisms. Rumen microorganisms consist mainly of obligatory anaerobes (bacteria, protozoa, archaea, and fungi), and bacteria represents the highest proportion of the microbial population (5). Previous research has shown that ruminants are born without bacteria in the rumen, but microorganisms in the environment will soon enter and colonize it (6). Rumen microbial fermentation and degradation of plant fibers is a complex and coordinated process; the feed is converted into digestive compounds, particularly volatile fatty acids (VFAs) and microbial proteins. Rumen microbiota are the link between ruminants and their diets, playing a key role in ruminant nutrition (7, 8), food digestion (9), physical development, energy balance (10), immunity regulation (8), and pathogen resistance (11). Previous research suggests that changes in rumen microbiota can alter its function (12); however, there are relatively few studies on how the composition and function of rumen microbes respond to extremely cold seasons.

The Qinghai-Tibet Plateau (QTP) has the highest altitude and the largest area of continuous grazing grassland in the world, accounting for 44% of China's 40 million hectare grasslands, and maintains ~20 million heads of yaks (5) and 50 million heads of Tibetan sheep (13). Forage mass and quality are strongly restricted by the great temperature difference between summer and winter in the QTP (3), resulting in approximately 37% nutrient-deficient livestock in northeastern QTP annually; this ultimately determines the productivity of livestock through rumen microbes and nutrient physiology, to a certain extent. In traditional ranches in the QTP, Tibetan sheep (*Ovis aries*) graze on natural pastures at >3,000 m above sea level without supplementary feeding, and are well adapted to the harsh high altitude environment, such as the lower temperature and risk of hypoxia (13). Tibetan sheep can adapt to extreme environments, including physiological adaptations, such as developed heart and molecular regulation mechanisms; for example, mutations in EPAS1 can enhance the average red blood cell hemoglobin concentration and average red blood cell volume (14). However, there is still a lack of in-depth research on the adaptability of Tibetan sheep based on rumen microorganisms. To explore the adaptation of Tibetan sheep to extremely cold seasons, we aimed to assess the differences in growth performance, forage digestion, serum biochemical indices, and rumen microbial communities of Tibetan sheep between summer and winter. We hypothesized that the decline in the nutritional quality of forage in the winter would cause a decrease in the digestibility of forage, serum immunity, and antioxidant status, which would ultimately lead to a reduction in growth performance. Our results show that in Tibetan sheep, the composition and function of rumen microorganisms are adjusted in different seasons, which might contribute to the animals' adaptation to extremely cold climates. The research results have great significance in explaining the survival adaptability of Tibetan sheep in different seasons on the QTP.

## Materials and Methods

### Study Site, Animals, and Experimental Diets

The study site was located in the QTP base of Lanzhou University in Maqu County, Gansu Province (33°404′N, 101°5212′E) in the northeastern part of the QTP, with an elevation of 3,700 m asl (15). The average temperature and relative humidity during the experimental period in the summer (July 1 to August 23, 2019) was 12.6°C and 72%, and the corresponding values were −2.5°C and 46%, respectively, during the experimental period in the winter (November 1 to December 24, 2019). All trial procedures were approved by the Animal Ethics Committee of the Lanzhou University (file No: 2010-1 and 2010-2). Twelve 24-month-old Tibetan sheep (38 ± 1.46 kg summer liveweight and 38 ± 1.32 kg winter liveweight; mean ± SD) were selected from a local herdsman in each season. Before the start of the experiment, the animals were ear-tagged, and drenched. The Tibetan sheep were fed in individual metabolic cages (1.0 × 1.5 m) with a water tank and a feed trough. They were fed for 54 days, which included 14 days of adaptation to the forage and conditions and a 40-day trial. They were fed 800 g dry matter (DM)/day (~2% of BW/days), three times per day, at 08:00, 12:00, and 18:00. Forage was collected daily in the morning; it consisted of natural pasture from 30 ha of a fenced alpine meadow from a native pasture, including grasses, sedges, and forbs. The species and proportions of the forage are listed in Supplementary Table 1. During the experiment, Tibetan sheep had no supplementary feed and had free access to fresh water.

### Forage Intake and Digestibility

During the formal experimental period, were performed accurate recording of the amount of forage before feeding and the amount of leftover feed after feeding to calculate the DM intake by experimental animals; then, 100 g of forage was sampled. The digestibility trial consisted of 7 days for sampling to determine the apparent digestibility of forage nutrients from days 33–39 of the experimental period, and daily feed intake and feces excretion were recorded. Before the morning feeding, the feces samples were collected, weighed, and mixed, and samples (100 g each) were mixed with 10 mL of 10% hydrochloric acid and stored in a plastic bag. The forage and feces samples were oven dried (60°C, 48 h), ground (<1 mm), and sieved before chemical analysis. DM, organic matter (OM), and crude fat ether extract (EE) were measured according to the methods described by the AOAC (16). The Kjeldahl method was used to determine the nitrogen content in the forage, and the crude protein (CP) content of the forage was calculated as 6.25 × N. The content of acid detergent fiber (ADF) and neutral detergent fiber (NDF) were measured and determined using the Van Soest method (17). Sodium sulfite (10 g/L of NDF solution) was added to the solution but without heat-stable α-amylase. Nutrient apparent digestibility was calculated from contents in the forage intake and the contents in fecal output.

### Growth Performance

To evaluate growth performance, the animals were weighed at the beginning (after 6 h starvation) and at the end (2 h before feeding in the morning) of the experimental period of each season. The average daily gain (ADG) changes were calculated based on the difference between the final and initial weights.

### Blood Samples

Jugular blood samples were collected into 10 mL evacuated collection tubes without anticoagulant. The collection was performed before morning feeding on day 40 of the experimental period. The samples were centrifuged (5,000 × *g*, 20 min, 4°C); the serum was then collected and frozen at −20°C for subsequent analysis. The concentrations of serum glucose (GLU), albumin (ALB), total protein (TP), globulin (GLO), superoxide dismutase (SOD), glutathione peroxidase (GSH-PX), malondialdehyde (MDA), and total antioxidant capacity (T-AOC) were analyzed using commercial kits (Model 100T/96S, Suzhou Keming Biotechnology Co. Ltd. China). Serum immunoglobulin (Ig) A, M, and G concentrations were measured using a microplate reader (Keda Biotechnology, Shanghai, China). Serum growth hormone (GH) levels were tested using commercial kits (Model 100T/96S, Jiangsu, China).

### VFA Profile Measurement and DNA Analysis

Rumen fluid samples (~50 mL) were collected 2 h after morning feeding using an oral stomach tube (5) on day 40 of the experimental period. Between the collection of samples, the oral tube was thoroughly cleaned with clean water, and the first 50 mL samples of each yak will be discarded to ensure that it is not contaminated by previous animals and its own saliva. The sample pH was immediately measured using a pH meter (Model 206-pH2, Testo, Germany), as previously described (5, 9, 12). The rumen-fluid samples were used for VFA testing, ammonia nitrogen (NH_3_-N) concentration analysis, and DNA extraction. For the analysis of ruminal VFA concentrations, the filtrate was thawed and centrifuged at 1,000 × *g* for 15 min and then analyzed by gas chromatography (GC-MS522; Wufeng Instruments, Shanghai, China) as described by Fan et al. (5). The NH_3_-N concentration was determined by colorimetry (UV-VIS8500, Tianmei, Shanghai, China) as described by Chaney and Marbach (18).

Total DNA was extracted from 2 mL of rumen liquid using the E.Z.N.A. DNA kit (Qiagen, Hilden, Germany). The V3-V4 hypervariable regions of the bacterial 16S rRNA gene were amplified using a thermocycler PCR system (GeneAmp 9700, Applied Biosystems, Foster City, CA, USA) and the following universal primers: forward (338F) 5′-ACTCCTAGGGAGGCAGCAG-3′; reverse (806R) 5′-GGACTACHVGGGTWTCTAAT-3′ (19). PCR amplification for next-generation sequencing (high-throughput sequencing) has been previously described (12). The 18 samples (12 samples from summer and 6 samples from winter) and the 6 samples (winter) were sequenced, respectively. Sequences were sorted based on their unique barcodes, followed by the removal of barcodes and primer sequences using QIIME (version 1.9.0; http://qiime.org). Raw tags were merged using FLASH (version 1.2.11) with default parameters (20). Low-quality reads were eliminated using QIIME (version 1.7.0) (21). Clean tags were compared to the Gold database using the UCHIME algorithm to eliminate chimera sequences. The sequences that possibly came from the mitochondrion and Chloroplast of the forage were moved. Effective tags were obtained for further analysis. These effective tags were clustered into operational taxonomic units (OTUs) with ≥97% similarity using UPARSE (version 7.0; http://drive5.com/uparse/) (22). Based on the SILVA (SSU123) database, the RDP classifier (version 2.2; https://sourceforge.net/projects/rdp-classifier/files/rdp-classifier/) was used to classify the representative sequences. Alpha diversity analysis including Chao1, Shannon, PD_whole_tree, and observed_species were calculated with QIIME (version 1.9.0). Principal coordinates analysis (PCoA) was used to compare treatments based on the weighted Uni-Frac distance metric (23).

### Statistical Analysis

The difference of growth performance, forage nutrient digestion, serum biochemical indexes, VFAs and alpha diversity of Tibetan sheep between seasons was compared using an independent sample t test based on SAS (SAS, version 9.2; SAS Institute Inc., Cary, NY, USA). Differences were considered statistically significant at *P* < 0.05. Microbial networks were generated to calculate the correlations between predominant taxa using Gephi software (version 0.9.2 https://gephi.org), and keystone taxa in the microbial communities were identified using the combined score of high mean degree, high closeness centrality, and low betweenness centrality (24). Pearson correlation coefficients between the relative abundances of the rumen bacteria (genus) and short chain fatty acids were calculated using the heatmap package in R software (version 4.0.2 https://CRAN.R-project.org). Structural equation model performed in the ‘SEM’ package of R was used to estimate the effect of seasons on forage nutrient compositions, rumen fermentation parameters, serum biochemical indexes, and microbial community diversity. The rumen microbiota functional pathways were predicted using PICRUSt2 (PICRUSt2 v2.3.0_b; https://github.com/picrust/picrust2) software based on 16S sequencing data (https://github.com/picrust/picrust2/wiki). PICRUSt2 was used to study the prediction of the bacterial community function in the rumen of Tibetan sheep in two seasons, and the difference in the abundance of KOs (KEGG orthology groups) in KEGG (Kyoto Encyclopedia of Genes and Genomes) level 2 between summer and winter were determined.

## Results

### Nutrient Composition of Forage

The chemical composition of the forage in the summer and winter is presented in Table 1. The contents of CP (*P* < 0.01) and EE (*P* < 0.01) were higher in the summer than in the winter. The opposite was found for the levels of NDF (*P* = 0.013) and ADF (*P* < 0.01). There were no differences in DM (*P* = 0.921) and OM (*P* = 0.767) between the two seasons.

**Table 1:** Common forage nutrient composition in the summer and winter (% of dry matter).

**Chemical composition**	**Season**		**SEM**	***p***
	**Summer**	**Winter**		
DM	38.36	38.61	0.8562	0.9214
OM	89.29	89.09	0.3346	0.7672
CP	12.27	6.38	0.6672	<0.01
EE	1.85	1.20	0.0718	<0.01
NDF	48.97	53.62	0.9742	0.0131
ADF	27.63	32.04	0.7512	<0.01

*DM, dry matter; OM, organic matter; CP, crude protein; EE, ether extract; NDF, neutral detergent fiber; ADF, acid detergent fiber; SEM, standard error of the mean*.

### Serum Profiles of Tibetan Sheep

The serum profile variables of Tibetan sheep in the summer and winter are presented in Table 2. The concentrations of GH (*P* = 0.002), TP (*P* = 0.037), ALB (*P* = 0.046), SOD (*P* < 0.01), GSH-PX (*P* = 0.014), IgG (*P* < 0.01), and IgM (*P* = 0.043) were higher in the summer than in the winter. However, there was no difference in serum GLO (*P* = 0.066), GLU (*P* = 0.069), MDA (*P* = 0.727), T-AOC (*P* = 0.746), and IgA (*P* = 0.134) between the two seasons.

**Table 2:** Comparison of serum biochemical indexes influenced by the summer and winter.

**Chemical composition**	**Season**		**SEM**	***p***
	**Summer**	**Winter**		
GH (ng/mL)	31.42	26.18	0.9926	0.0019
TP (g/L)	77.61	64.86	3.1762	0.0370
ALB (g/L)	31.48	26.35	1.2842	0.0463
GLO (g/L)	46.83	38.72	2.1421	0.0656
GLU (g/L)	3.74	2.58	0.1686	0.0691
MDA (nmol/mL)	0.86	0.79	0.1133	0.7272
SOD (U/ml)	225.38	113.26	15.6452	<0.01
T-AOC (U/ml)	6.58	6.47	0.2511	0.7464
GSH-PX (μmol/L)	960.53	840.28	12.4374	0.0143
IgA (μg/mL)	0.76	0.58	0.0614	0.1336
IgG (μg/mL)	31.57	18.65	1.8392	<0.01
IgM (μg/mL)	1.84	1.26	0.1411	0.0433

*GH, growth hormone; TP, total protein; ALB, albumin; GLO, globulin; GLU, glucose; MDA, malondialdehyde; SOD, superoxide dismutase; T-AOC, total antioxidant capacity; GSH-PX, Glutathione peroxidase; IgA, immunoglobulin A; IgG, immunoglobulin G; IgM, immunoglobulin M; SEM, standard error of the mean*.

### Nutrient Intake and Apparent Digestibility in Tibetan Sheep

ADG was significantly higher in the summer than in the winter (*P* < 0.01; Table 3). The intake of DM (*P* < 0.01), CP (*P* < 0.01), and EE (*P* < 0.01) was also higher in the summer than in the winter. However, the intake of NDF (*P* < 0.01) and ADF (*P* < 0.01) was higher in the winter than in the summer. There was no difference in OM (*P* = 0.056) between the two seasons. Apparent digestibility of DM (*P* < 0.01), OM (*P* = 0.010), CP (*P* < 0.01), NDF (*P* < 0.01), ADF (*P* < 0.01), and EE (*P* < 0.01) was higher in the summer than in the winter.

**Table 3:** Effect of season on average daily gain, dry matter intake, and apparent digestibility of nutrients.

**Chemical composition**	**Season**		**SEM**	***p***
	**Summer**	**Winter**		
BW (kg)	38.15	38.13	0.9982	0.2213
ADG (g/d)	96.28	−50.39	22.1251	<0.01
**DM Intake (g/d, DM basis)**				
DM	797.84	741.42	11.6083	<0.01
OM	716.85	672.94	10.3785	0.0561
CP	97.72	47.39	7.1761	<0.01
NDF	387.83	397.55	1.7514	<0.01
ADF	217.82	236.09	3.7826	<0.01
EE	14.82	10.07	0.6132	<0.01
**Apparent digestibility (% DM)**				
DM	65.69	55.89	1.8472	<0.01
OM	69.82	57.48	2.6285	0.0102
CP	66.41	40.13	4.1996	<0.01
NDF	64.45	54.26	2.0927	<0.01
ADF	61.10	51.48	1.9552	<0.01
EE	49.71	41.70	1.6531	<0.01

*ADG, average daily gain; DM, dry matter; OM, organic matter; CP, crude protein; NDF, neutral detergent fiber; ADF, acid detergent fiber; EE, ether extract; SEM, standard error of the mean*.

### Rumen Fermentation Parameters

The rumen fermentation parameters for Tibetan sheep in the summer and winter are presented in Table 4. The concentrations of rumen NH_3_-N (*P* < 0.01) and TVFA (*P* < 0.01) and the proportion of propionate (*P* = 0.031), butyrate (*P* = 0.024), and valerate (*P* < 0.01) were higher in the rumen of Tibetan sheep in the summer than in the winter. The proportion of acetate (*P* < 0.01) and the ratio of acetate to propionate were higher in the winter than in the summer.

**Table 4:** Rumen fermentation parameters in Tibetan sheep.

**Chemical composition**	**Season**		**SEM**	***p***
	**Summer**	**Winter**		
pH	6.43	6.81	0.041	0.037
NH_3_-N (mg/dL)	10.03	4.26	0.751	<0.01
TVFA (mmol/L)	54.70	46.56	1.455	<0.01
Acetate (%)	71.4	77.98	1.195	<0.01
Propionate (%)	15.71	12.32	0.824	0.031
Butyrate (%)	9.98	7.56	0.523	0.024
Isobutyrate (%)	1.26	1.09	0.069	0.254
Valerate (%)	0.88	0.61	0.049	<0.01
Isovalerate (%)	0.75	0.92	0.041	0.052
Acetate/propionate	4.58	7.17	0.511	0.004

*NH_3_-N, ammonia nitrogen; TVFA, total volatile fatty acids; SEM, standard error of the mean*.

### Microbial Community Composition

**Figure 1:**
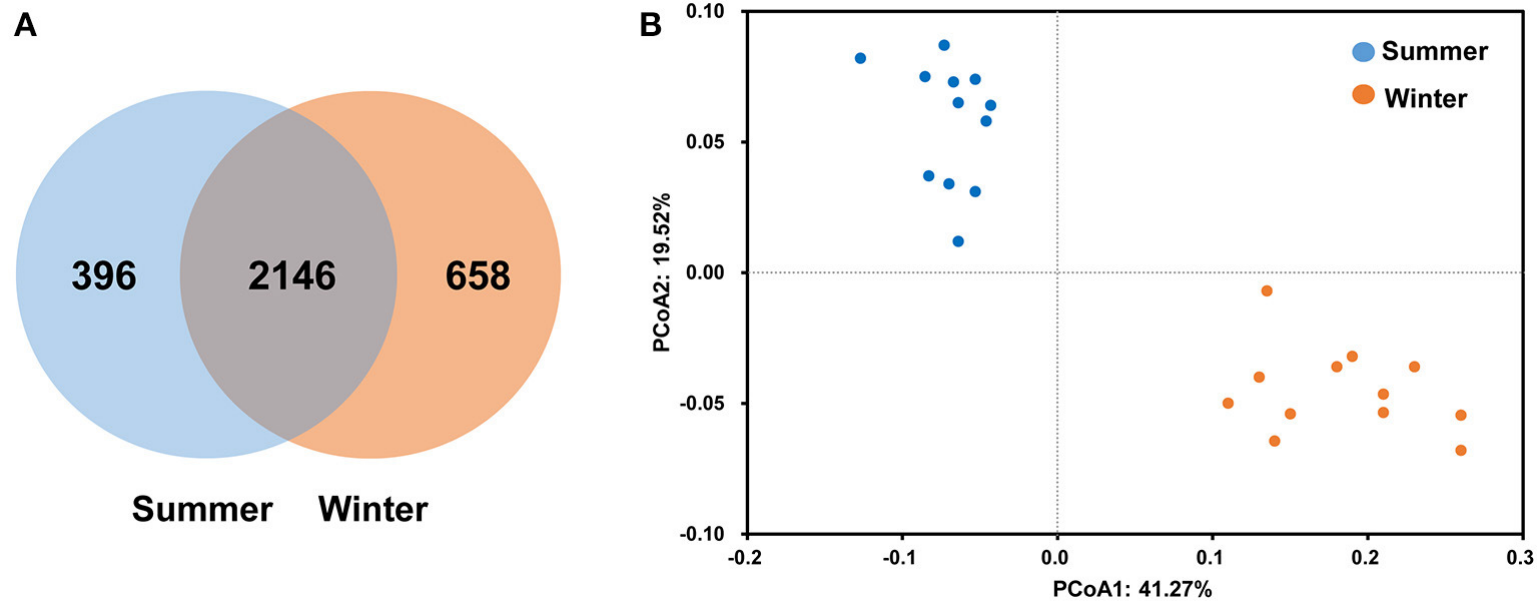
Differences in community dissimilarities and operational taxonomic units (OTUs) between summer and winter. Venn diagram **(A)** indicates specific and shared OTUs in both seasons. The weighted UniFrac distance **(B)** was used to calculate the differences in Tibetan sheep rumen microbiota in the different seasons, and principal coordinate analysis (PCoA) was used to calculate the coordinates.

**Figure 2:**
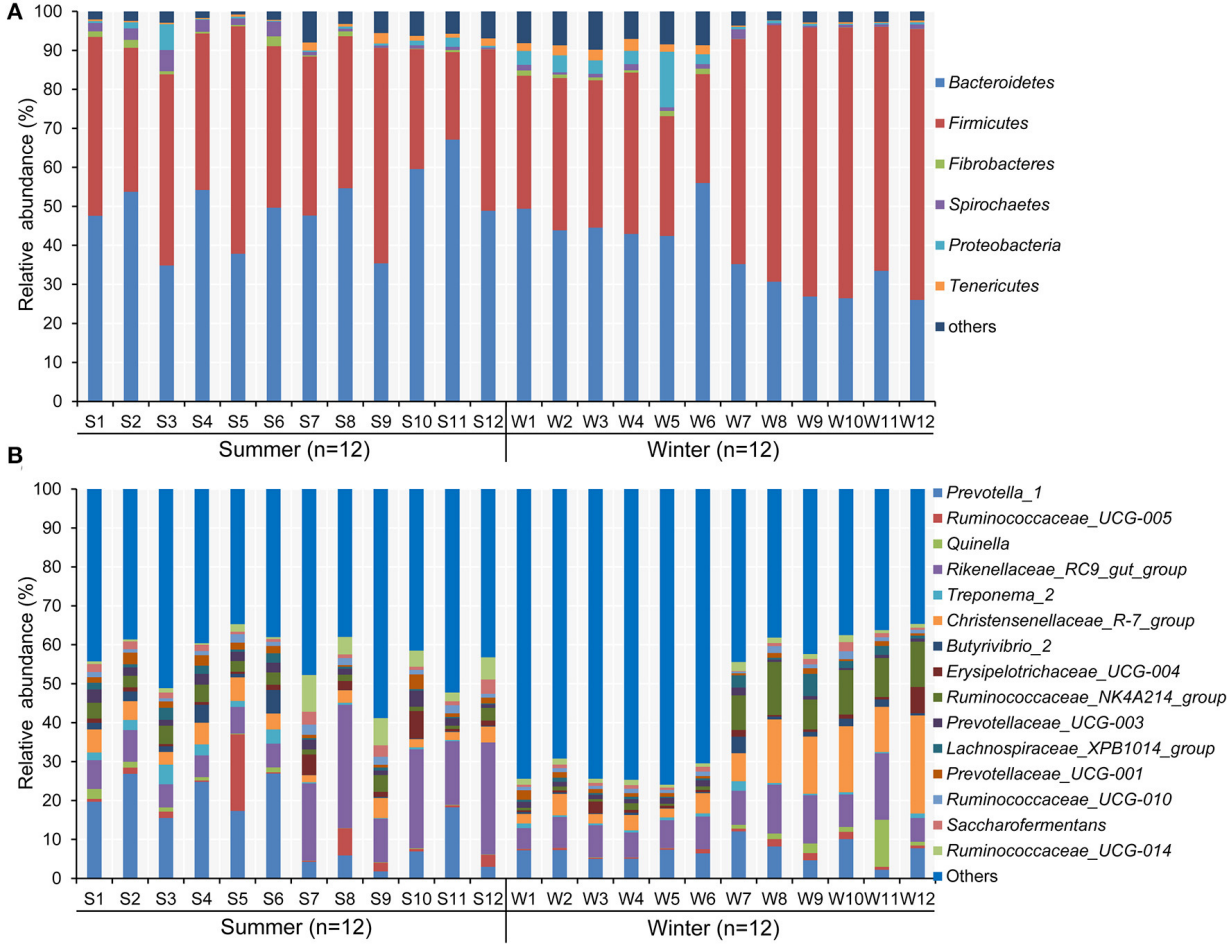
Composition of bacterial communities in Tibetan sheep at **(A)** phylum and **(B)** genus levels in the summer and winter. Only taxa with an average relative abundance >0.5% are displayed.

**Figure 3:**
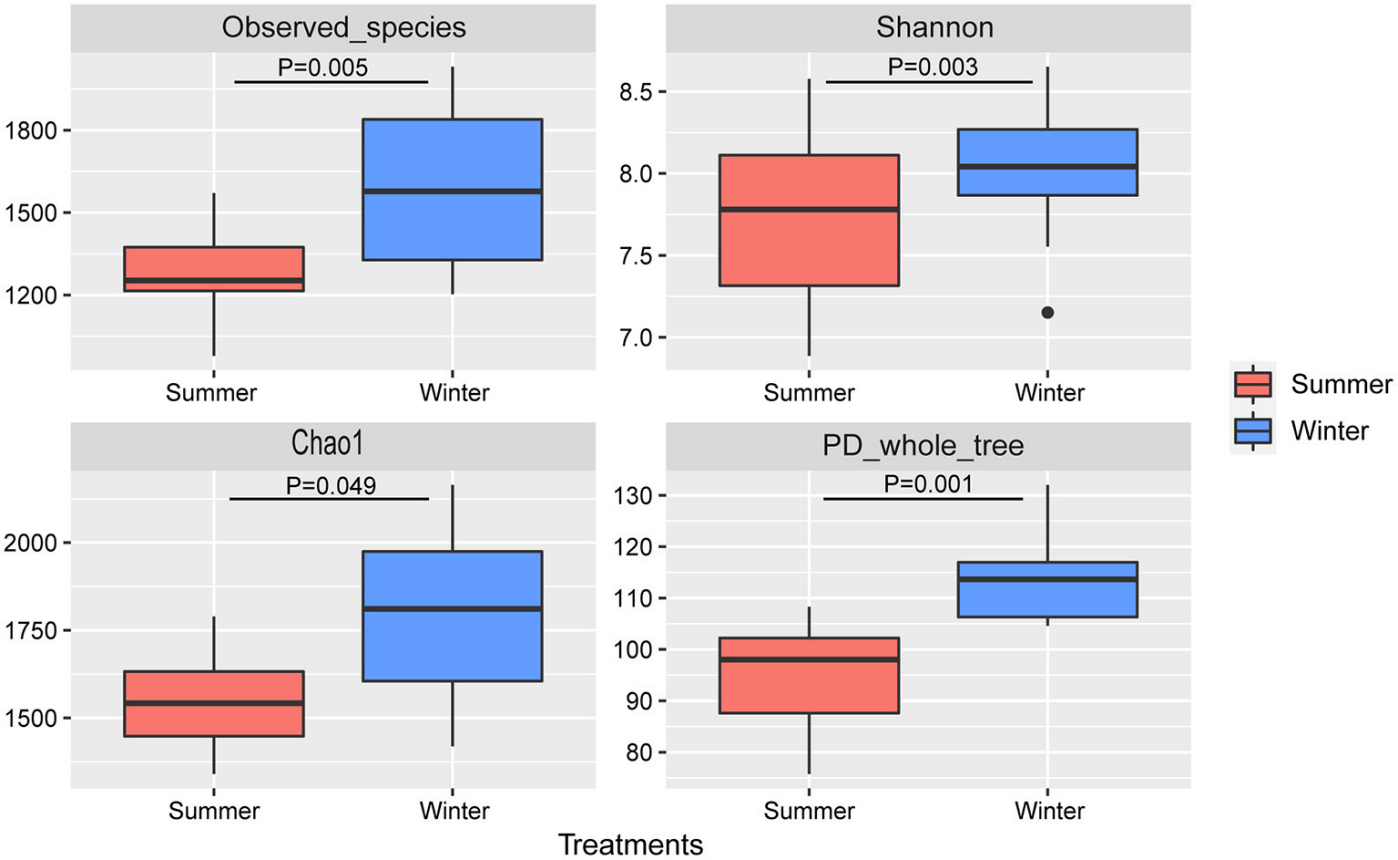
Microbial community diversities in the summer and winter. A significant difference is indicated by *P* < 0.05.

According to the Venn diagram, 2,146 OTUs in the rumen of Tibetan sheep were common to both seasons, and there were 396 and 658 unique OTUs in the summer and winter, respectively (Figure 1A). According to the PCoA (Figure 1B), obvious differences in the microbial communities in the different seasons were observed. Taxonomic analysis of the reads revealed 24 bacterial phyla. Bacteroidetes and Firmicutes were the predominant phyla, accounting for 43.73 and 41.36% of the relative abundances, respectively (Figure 2A); these were followed by Proteobacteria, Spirochaetes, and Tenericutes, representing 2.07, 1.49, and 1.18% of the relative abundances, respectively. At the genus level, 230 taxa were identified. *Rikenellaceae_RC9_gut_group* (11.75%) was the most dominant genus, followed by *Prevotella_1* (10.62%), *Christensenellaceae_R-7_group* (6.75%), *Ruminococcaceae_NK4A214_group* (4.31%), *Ruminococcaceae_UCG-014* (2.24%), and *Ruminococcaceae_UCG-005* (2.03%) (Figure 2B). The community richness estimates (Chao 1 estimator; *P* = 0.049), diversity indices (Shannon index; *P* = 0.003), Observed_species (*P* = 0.005), and PD_whole_tree (*P* = 0.001) were significantly higher in the winter than in the summer (Figure 3).

The effects of season on the prevalence of certain bacterial phyla and genera (average relative abundance >0.5% in one group) in the rumen of Tibetan sheep are presented in Supplementary Tables 2, 3, respectively. At the phylum level, the relative abundance of Bacteroidetes (49.28% in the summer vs. 38.17% in the winter, *P* = 0.011) was higher in the summer than in the winter, whereas the relative abundance of Firmicutes (32.31% in the summer vs. 50.41% in the winter, *P* = 0.014) and the ratio of Firmicutes to Bacteroidetes (0.66 in the summer vs. 1.51 in the winter, *P* = 0.004) were higher in the winter than in the summer. At the genus level, the relative abundance of *Prevotella_1* (14.29% in the summer vs. 6.95% in the winter, *P* = 0.017), *Prevotellaceae_UCG-003* (2.24% in the summer vs. 1.12% in the winter, *P* = 0.001), *Ruminococcus_1* (0.87% in the summer vs. 0.47% in the winter, *P* = 0.044), *Saccharofermentans* (1.77% in the summer vs. 0.95% in the winter, *P* = 0.025), and *Ruminococcaceae_UCG-014* (3.17% in the summer vs. 1.30% in the winter, *P* = 0.043) were relatively higher in the summer than in the winter, whereas that of *Christensenellaceae_R-7_group* (4.01% in the summer vs. 9.48% in the winter, *P* = 0.020) presented the opposite pattern.

### Network Analysis of Bacterial Communities

**Figure 4:**
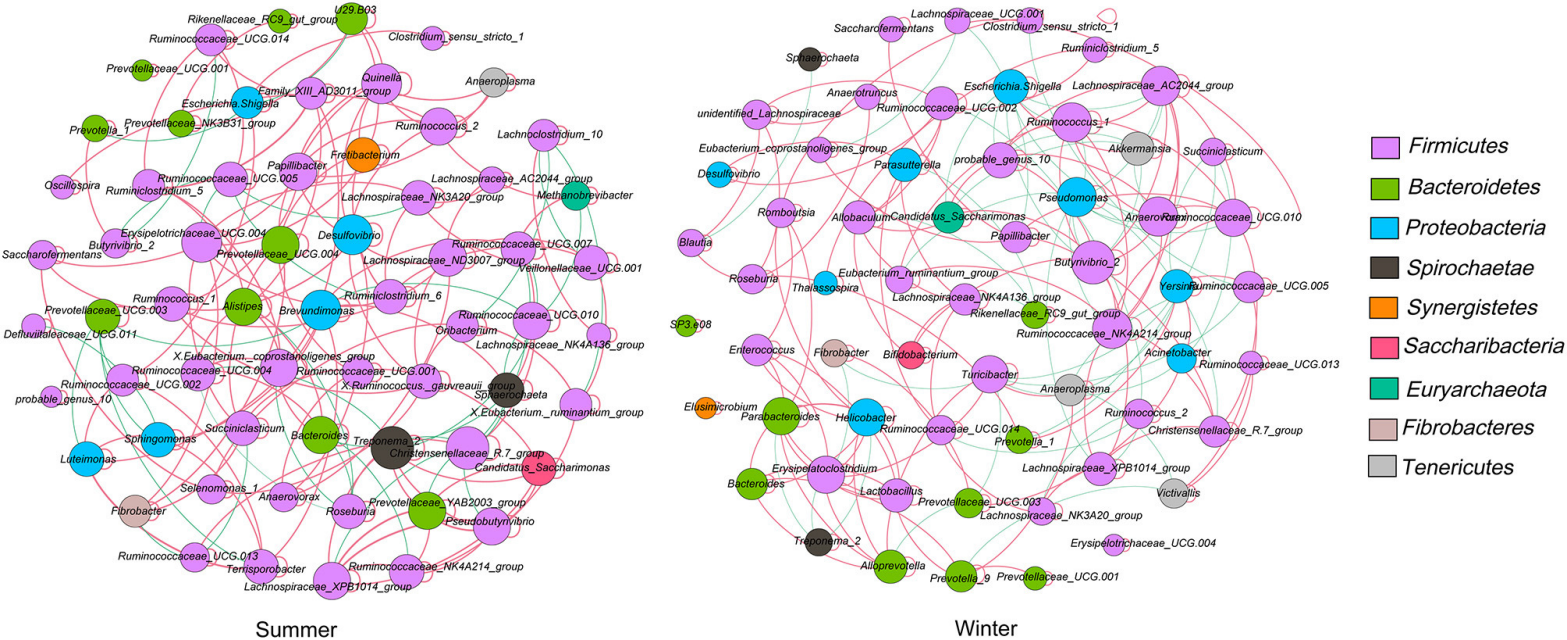
Interaction networks of the rumen microbiota. 16S rRNA gene-based correlation network of the rumen microbiota, displaying statistically significant interactions with absolute value of correlation coefficients > 0.6. The node size was scaled based on the overall abundance of each taxa in the microbiota. A red edge indicates a positive correlation and green edge indicates a negative correlation.

The microbial network was used to analyze the microbial interactions among rumen bacterial communities of Tibetan sheep and to statistically identify bacterial genera that are keystone taxa that regulate the fermentation process. The results showed that the season changed the correlation within the microbiota (Figure 4). We verified that the negative correlations in the winter were stronger than those in the summer. The putative drivers of keystone taxa in the rumen microbial communities of Tibetan sheep from the two seasons were estimated using the combined score of the high mean degree, high closeness centrality, and low betweenness centrality (Supplementary Table 4). The results indicated that *Prevotellaceae_YAB2003_group, Lachnospiraceae_XPB1014_group*, and *Pseudobutyrivibrio*—in the summer—and *Ruminococcus_1, Lachnospiraceae_AC2044_group*, and *Anaerovorax*—in the winter— could be considered keystone taxa in the rumen of Tibetan sheep.

### Correlations Between Forage Nutrient Compositions, Rumen Fermentation Parameters, Serum Biochemical Indexes, and Bacterial Community Diversity

**Figure 5:**
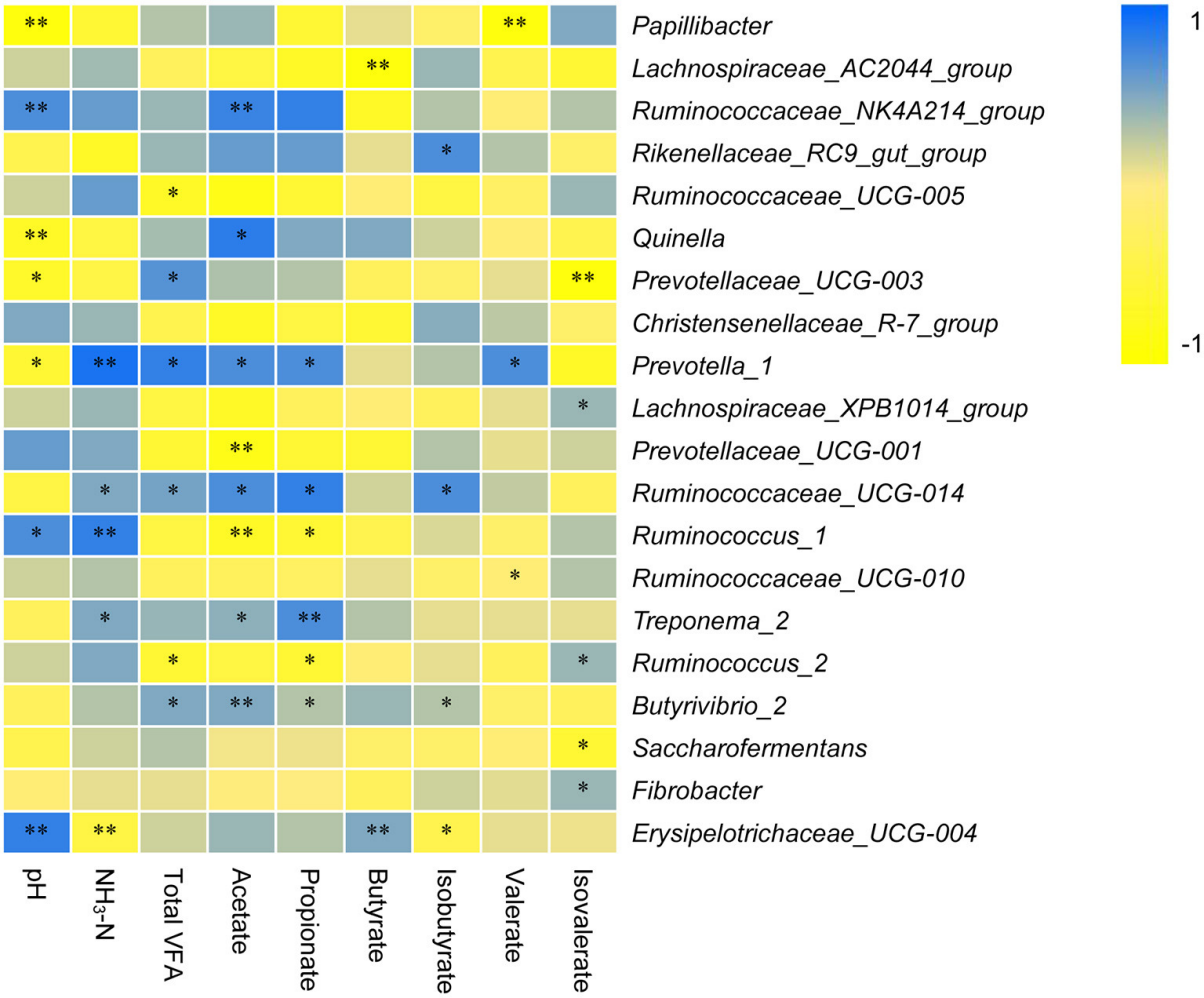
Relationship among NH_3_-N, bacterial Communities, and short-chain fatty acids (SCFAs). ** and * indicate significance levels at 0.01 and 0.05, respectively.

The correlation between the dominant rumen bacterial genera and pH, NH_3_-N, or TVFA is shown in Figure 5. The pH was positively correlated with the genera *Ruminococcaceae_NK4A214_group* (*r* = 0.705), *Ruminococcus_1* (*r* = 0.729), and *Erysipelotrichaceae_UCG-004* (*r* = 0.853) and negatively correlated with *Papillibacter* (*r* = −0.903), *Quinella* (*r* = −0.783), *Prevotellaceae_UCG-003* (*r* = −0.672), and *Prevotella_1* (*r* = −0.637). NH_3_-N was positively correlated with the genera *Prevotella_1* (*r* = 0.827), *Ruminococcaceae_UCG-014* (*r* = 0.527), *Ruminococcus_1* (*r* = 0.801), and *Treponema_2* (*r* = 0.538) and negatively correlated with *Erysipelotrichaceae_UCG-004* (*r* = −0.495). TVFA was directly correlated with *Prevotellaceae_UCG-003* (*r* = 0.684), *Prevotella_1* (*r* = 0.856), *Ruminococcaceae_UCG-014* (*r* = 0.581), and *Butyrivibrio_2* (*r* = 0.537) and negatively correlated with *Ruminococcaceae_UCG-005* (*r* = −0.706) and *Ruminococcus_2* (*r* = −0.638). Acetate was positively correlated with the genera *Ruminococcaceae_NK4A214_group* (*r* = 0.795), *Quinella* (*r* = 0.831), *Prevotella_1* (*r* = 0.793), *Ruminococcaceae_UCG-014* (*r* = 0.752), *Treponema_2* (*r* = 0.574), and *Butyrivibrio_2* (*r* = 0.504) and negatively correlated with *Prevotellaceae_UCG-001* (*r* = −0.893) and *Ruminococcus_1* (*r* = −0.745). Propionate was positively correlated with *Prevotella_1* (*r* = 0.753), *Ruminococcaceae_UCG-014* (*r* = 0.837), and *Treponema_2* (*r* = 0.746) and negatively correlated with *Ruminococcus_1* (*r* = −0.648) and *Ruminococcus_2* (*r* = −0.624). Butyrate was positively correlated with *Erysipelotrichaceae_UCG-004* (*r* = 0.503) and negatively correlated with *Lachnospiraceae_AC2044_group* (*r* = −0.943). Isobutyrate was positively correlated with *Rikenellaceae_RC9_gut_group* (*r* = 0.735) and *Ruminococcaceae_UCG-014* (*r* = 0.712) and was inversely correlated with *Erysipelotrichaceae_UCG-004* (*r* = −0.427). Valerate was positively correlated with *Prevotella_1* (*r* = 0.742) and negatively correlated with *Papillibacter* (*r* = −0.906). Isovalerate was positively correlated with the genera *Lachnospiraceae_XPB1014_group* (*r* = 0.493), *Ruminococcus_2* (*r* = 0.472), and *Fibrobacter* (*r* = 0.407) and negatively correlated with *Prevotellaceae_UCG-003* (*r* = −0.915) and *Saccharofermentans* (*r* = −0.673).

The correlation analysis among dominant rumen bacterial genus, forage nutrient compositions, rumen fermentation parameters, and serum biochemical indexes were investigated in summer and winter (Supplementary Figure 1). Overall, the interaction structure of the summer was more complex than that in winter. In addition, we found that *Prevotella_1* was negatively corrected with DM in summer, while positively corrected with EE in winter. *Prevotellaceae_UCG-003* was positively corrected with TVFA, GSH-PX, IGG and GH, and negatively corrected with ADF in summer, while *Prevotellaceae_UCG-001* was the dominant bacteria in winter and just negatively corrected with GLU and IGM. In addition, we estimated the relationships among the season, forage nutrient compositions, rumen fermentation parameters, serum biochemical indexes, and bacterial community diversity based on structural equation model (SEM) analysis. Our results showed that season may directly influence forage nutrient composition, rumen fermentation parameters, and serum biochemical indexes and indirectly shape rumen bacterial alpha diversity (Shannon index) by regulating forage nutrient compositions and serum biochemical indexes (Supplementary Figure 2).

### PICRUSt2 Gene Function Estimation

**Figure 6:**
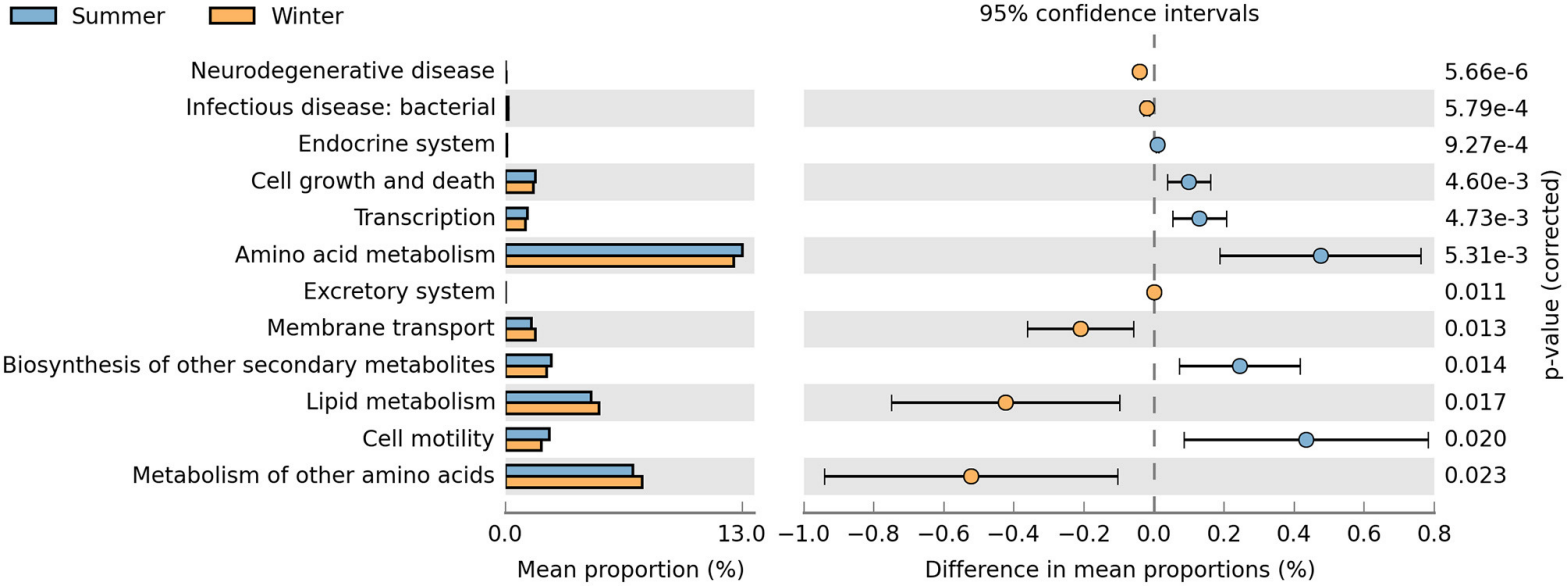
Relative abundance of predicted functions and significance of KOs (KEGG orthology groups) at KEGG (Kyoto Encyclopedia of Genes and Genomes) level 2 between the summer and winter. Student's *t*-tests were used to compare the abundance changes two groups; only differences with a *P* < 0.05 are reported.

KOs in level 2 (Figure 6) suggested that the pathways related to cell growth and death, transcription, amino acid metabolism, membrane transport, biosynthesis of other secondary metabolites, lipid metabolism, and cell motility were enriched differently between the summer and winter groups. The relative abundances of cell growth and death, transcription, amino acid metabolism, biosynthesis of other secondary metabolites, and cell motility terms were significantly higher in the summer than in the winter (*P* < 0.05). Conversely, the relative abundances of membrane transport and lipid metabolism terms were significantly higher in the winter than in the summer (*P* < 0.05).

## Discussion

The seasonal availability and quality of forage might have affected the performance of Tibetan sheep, corroborating the results of a previous study (2). The species and season of plants are important factors that affect forage quality (3). The results of this study showed that the CP content of forage grown in the summer was significantly higher than that in the winter, whereas the opposite was found for the fibrous fraction contents. This could be due to the low ratio of forage leaves to stems in plants in the winter, which would affect the intake of forage and hence reduce the forage DMI by the Tibetan sheep compared with the results of other studies (25, 26). Furthermore, the combined effects of low CP and high NDF and ADF contents of winter forage resulted in decreased ADG in Tibetan sheep. The forage nutritional CP fraction is the most important nutrient required by livestock. If dietary CP falls below 7.0%, rumen microbial activity will be suppressed and forage intake will be reduced (27). Moreover, the CP content of forage in this study was >7% in the summer, which consequently improved the ADG in Tibetan sheep. Ephrem et al. (27) reported that the nutritional values, including CP, NDF, and ADF contents, were higher in the summer than in the winter, affecting feed intake and the production efficiency of sheep grazing on the QTP. In this context, our results were in agreement with the work reported by Xue et al. (10).

GH is a peptide that is essential for stimulating the production and growth of IGF-1 (28). In this study, GH concentrations were higher in the serum of Tibetan sheep in the summer than in the winter. ALB is an important protein source for liver synthesis, and its main functions include providing energy, repairing tissues, and acting as a transport carrier of nutrients to maintain the dynamic balance of tissue proteins (29). In our study, the concentrations of ALB were higher in the serum of Tibetan sheep in the summer than in the winter, indicating that in the winter, the forage was deficient in essential nutrients. SOD is not only an enzyme that removes superoxide anions but also produces high amounts of H_2_O_2_, and it plays an important role in biological antioxidant systems (28); GSH-Px is an important indicator of antioxidant activity in livestock (30). In the present study, the concentrations of SOD and GSH-PX were higher in the serum of Tibetan sheep in the summer than in the winter, indicating that Tibetan sheep might have improved antioxidant capacity in the summer. Similarly, the concentrations of IgG and IgM, indicators of nonspecific humoral immunity in ruminants, were also higher in the summer than in the winter (28).

A previous study showed that forage presented higher levels of nutrients and were more digestible in the summer, when they were succulent and growing rapidly, than in the winter (31). This result was consistent with our findings in which the digestibility of forage decreased as the forage matured. This indicates that the nutrient quality of forage influences nutrient digestibility. An increase in forage NDF and ADF content resulted in linearly decreased CP digestibility because of the reduction in non-structural carbohydrates, and this consequently limited the supply of fermentable energy for rumen microorganisms to degrade dietary protein for their growth (3). This result corroborates our findings in which CP digestibility was lower in the winter than in the summer. Yang et al. (3) suggested that forage fiber content (NDF and ADF) would correlate negatively with the digestibility of all plant material. In this investigation, NDF and ADF contents were negatively correlated with their digestibility.

Ordinarily, ecosystems with high species diversity will be stable and show high levels of performance and function (32, 33). Although there are different interactions between species in different ecosystems, there is evidence that high species diversity provides more functional redundancy and buffers ecosystem functions to prevent the extinction of species (34). In the host-microbial system, different bacterial communities can contribute to a unique set of digestive enzymes to improve forage processing and digestion (35); thus, high microbial diversity is usually related to strong metabolic capacity and stability. A previous study showed that the rumen microbiota diversity of Tibetan sheep improves the fermentation efficiency of forage fiber and expedites the stability of the rumen microbial ecosystem (36). In the present study, higher rumen microbial diversity was found in the winter than in the summer, indicating that the rumen microbial community of Tibetan sheep might have enhanced ability to use high-fiber forage to help them meet their energy requirements in cold and harsh habitats during the winter (12). In addition, the predicted gene functional profiles of the rumen microbiota of Tibetan sheep showed that most of these genes were related to membrane transport and cell metabolism (e.g., lipid and protein metabolism) and were overrepresented in the winter. This result implies that Tibetan sheep improve metabolic functions of rumen microbiota to cope with the low nutritional quality of the forage in the winter (37). However, our results only predict genomic information and might not represent the true function of Tibetan sheep rumen bacteria. Therefore, it is necessary to further use metagenomics to study the role of these genes in the adaptation of animals to extreme environments.

In this study, Bacteroidetes and Firmicutes were the most predominant bacterial phyla in the rumen of Tibetan sheep, corroborating the results of previous studies on Tibetan sheep (38, 39), cattle (40), sheep (41), yaks (5, 9, 12), goats (42), and pikas (43), and indicating that these bacteria play an important role in the ecology and function of the mammalian gastrointestinal tract. Kim et al. (44) conducted a meta-analysis of all selected 16S rRNA sequences stored in the NCBI database to summarize the distribution of rumen bacteria in major domestic animals and found that the proportions of Bacteroidetes and Firmicutes were approximately 31% and 56%, respectively. Interestingly, in the present study, the content of Bacteroidetes exceeded the mean proportion; however, the content of Firmicutes was lower than the mean proportion. A previous study showed that Firmicutes members are mainly responsible for energy conversion and harvesting (45), whereas Bacteroidetes members play an important role in carbohydrate degradation and protein hydrolysis (46). In this study, Bacteroidetes were more abundant in the summer, whereas Firmicutes were more abundant in the winter. The increase in the relative abundance of Firmicutes and the Firmicutes/Bacteroidetes ratio demonstrates that Tibetan sheep might exhibit improved energy utilization rates of forage and increased resistance to winter cold stress (12). Studies have confirmed that the ratio of Firmicutes/Bacteroidetes in goats and bovine is strong related to body fat storage and animal obesity (47–51).

At the genus level, *Ruminococcaceae_UCG-014* and *Ruminococcus_1*, which are related to plant cellulose fermentation, were more abundant in the summer than in the winter, demonstrating that these microorganisms might be involved in the degradation of forage (52). Importantly, the bacterial taxa *Prevotella_1* and *Christensenellaceae_R-7_group* in the rumen of Tibetan sheep were enriched in the winter, indicating that these microorganisms could help to adapt to harsh winter conditions such as low temperature and low oxygen (7, 53). The enrichment of these microorganisms in the rumen of winter-grazing sheep might be involved in the important functions of the host. For example, *Prevotella_1* degrades simple sugars, starches, and other polysaccharides as energy substrates to produce the glucogenic substrate succinate (53). The genus *Christensenellaceae_R-7_group* includes genes for critical hemicellulase and cellulase secretase, which could improve the ability of Tibetan sheep to degrade cellulose and obtain energy from indigestible polysaccharides (7). These genera in the rumen of Tibetan sheep can help improve host metabolic capacity and resistance to the low temperature and low oxygen environment in the winter. However, the ecological function of these bacteria in the rumen of Tibetan sheep requires further study. In the summer, *Saccharofermentans* were enriched in the rumen of Tibetan sheep, and these bacteria are considered VFA-producing (13), but as there is no known pure culture strain, the metabolic function of this genus is still unclear.

Rumen propionic acid production is enhanced and can lead to an increased concentration of glucose, as glucose can be produced by gluconeogenesis from propionic acid (29). Acetic acid production in the rumen is closely related to fiber degradation (54), and the values were higher in the winter than in the summer, which could be due to higher fiber degradation. Studies have shown that acetic acid in the rumen can significantly reduce the efficiency of energy utilization (28). In this study, the acetate:propionate ratio in the winter was enhanced, implying that the rumen profile shifted and the efficiency was reduced; however, in the summer, a lower acetate:propionate ratio and a higher TVFA concentration was verified, implying that the energy efficiency in the summer is higher than that in the winter. Some bacteria, including *Butyrivibrio_2, Prevotella_1, Treponema_2*, and *Ruminococcaceae_UCG-014*, were positively associated with the concentrations of acetate, propionate, and total VFA, indicating that these bacteria could be beneficial for VFA production. For example, *Butyrivibrio_2* might efficiently utilize fibrous and starchy substrates to produce butyrate (52); moreover *Prevotella_1* might utilize simple sugars and polysaccharides to produce propionate (55). However, because of the very complex interactions among bacteria, such as resource competition and cross-substitution, it is difficult to know which bacteria directly cause the production of a specific VFA (56). Through qPCR analysis, Liu et al. (36) found that the number of main cellulolytic and proteolytic bacteria in the rumen of grazing Tibetan sheep in the summer was significantly higher than that in the winter. This result was consistent with the higher nutrient digestibility and ADG of Tibetan sheep in the summer. Therefore, we hypothesize that in the warm season, a high abundance of rumen functional bacteria can improve the digestibility of forage and at the same time result in the production high concentrations of NH_3_-N and VFAs, which can quickly improve the growth performance of Tibetan sheep.

The vital role of the microbial consortium in ecosystem functions has been defined, and the relationships between microorganisms in the rumen fermentation ecosystem are very complex (57). There might be some species for which their location in the rumen fermentation ecosystem is not proportionate with their abundance (58). The current study is the first to identify the keystone taxa with network topological properties in the rumen of Tibetan sheep. The results demonstrate that the season alters the correlations among microflora and showed that the keystone genus is different between the two seasons. Network analysis could reveal species interactions from both positive and negative aspects (36). Negative interactions might weaken competitive relations, whereas positive interactions could strengthen competitive relations (59). In our study, the number of negative links in the winter was higher than that in the summer. We speculate that owing to the low nutritional quality of forage in the winter and lack of forage, microorganisms can make full use of the limited low-quality forage resources by strengthening cooperation. This further shows that season can regulate the microbial dynamics of the Tibetan sheep rumen.

## Conclusion

Our results indicate that rumen microorganisms of grazing Tibetan sheep have strong plasticity and might modulate function in response to environmental changes. In the summer season, the high relative abundance of Bacteroidetes, *Prevotella_1, Prevotellaceae_UCG-003, Ruminococcus_1, Saccharofermentans*, and *Ruminococcaceae_UCG-014* promotes forage degradation and fermentation to rapidly improve the growth performance and increase the serum immunity and antioxidant capacity of Tibetan sheep. In the winter season, the increase in Firmicutes, the Firmicutes/Bacteroidetes ratio, rumen microbial diversity, synergy between microorganisms, and metabolic pathways could enable Tibetan sheep to maximize the usage of low-quality forage to cope with the cold conditions.

## Data Availability Statement

The original contributions presented in the study are included in the article/Supplementary Material, further inquiries can be directed to the corresponding authors at Fujiang Hou;cyhoufj@lzu.edu.cn.

## Ethics Statement

All trial procedures strictly followed the rules and regulations of the Experimental Field Management protocols (File No: 2010-1 and 2010-2) of Lanzhou University and were approved by the Animal Ethics Committee of the University. Written informed consent was obtained from the owners for the participation of their animals in this study.

## Author Contributions

FH conceived and designed the experiments. XC and QF conducted animal experiments and sample collection. QF was responsible for data analysis and wrote the original manuscript. All authors have revised and approved the final manuscript.

## Conflict of Interest

The authors declare that the research was conducted in the absence of any commercial or financial relationships that could be construed as a potential conflict of interest.
