# Supplementary material for: Rumen Microbiota of Tibetan Sheep (Ovis aries) Adaptation to Extremely Cold Season on the Qinghai-Tibetan Plateau
Source: Front Vet Sci. 2021 May 25;8:673822. doi: 10.3389/fvets.2021.673822 (PMC8185353; doi:10.3389/fvets.2021.673822)
Supplement: Supplementary file 1 [file Data_Sheet_1.docx]

Supplementary Material

# Supplementary Tables

**Supplementary Table 1.** Main types and proportions of forage on the Qinghai-Tibetan Plateau in the summer and winter.

| Item | Major species communities in the grassland | Above-ground biomass/(g/m^2^) | High quality herbage/(g/m^2^) | Other herbage/(g/m^2^) |
| --- | --- | --- | --- | --- |
| Summer | *Elymus nutans*, *Poa pratensi*, *Kobresia humili*, *Potentill abifurca*, *Saussurea pulchra*, *Ajania tenuifolia* | 286.57 | 163.22 | 123.35 |
| Winter | *Elymus nutans*, *Poa pratensi*, *Kobresia graminifolia* | 43.83 | 28.67 | 15.16 |

The high quality herbage refers to the grasses which are classified as Sedges and Grasses, while the other herbage refers to the grasses which are not classified as Sedges and Grasses.

**Supplementary Table 2.** Composition of at the phylum level in the summer and winter (average relative abundance > 0.5%).

| Phylum | Season | | SEM | *p*-Value |
| --- | --- | --- | --- | --- |
|  | Summer | Winter |  |  |
| Bacteroidetes | 49.28 | 38.17 | 2.2653 | 0.0106 |
| Firmicutes | 32.31 | 50.41 | 3.8103 | 0.0139 |
| Fibrobacteres | 0.82 | 0.54 | 0.1446 | 0.3354 |
| Spirochaetes | 1.94 | 1.04 | 0.2567 | 0.0806 |
| Proteobacteria | 1.25 | 2.89 | 0.6294 | 0.2012 |
| Tenericutes | 0.99 | 1.37 | 0.2012 | 0.3651 |
| Firmicutes*/*Bacteroidetes | 0.66 | 1.51 | 0.1579 | 0.0043 |

SEM: standard error of the mean.

**Supplementary Table 3.** Composition of rumen bacterial communities of Tibetan sheep in the summer and winter (average relative abundance > 0.5%).

| Phylum | Genus | Season | | SEM | *p*-Value |
| --- | --- | --- | --- | --- | --- |
|  |  | Summer | Winter |  |  |
| Bacteroidetes | *Prevotella_1* | 14.29 | 6.95 | 1.5885 | 0.0171 |
|  | *Rikenellaceae_RC9_gut_group* | 14.44 | 9.05 | 1.5520 | 0.0823 |
|  | *Prevotellaceae_UCG-003* | 2.24 | 1.12 | 0.1855 | 0.0010 |
|  | *Prevotellaceae_UCG-001* | 1.71 | 1.09 | 0.1741 | 0.0749 |
| Firmicutes | *Lachnospiraceae_AC2044_group* | 0.76 | 0.42 | 0.1252 | 0.1790 |
|  | *Papillibacter* | 0.59 | 0.43 | 0.0850 | 0.3659 |
|  | *Ruminococcaceae_UCG-005* | 3.16 | 0.89 | 0.8195 | 0.1716 |
|  | *Quinella* | 0.67 | 1.62 | 0.5005 | 0.3527 |
|  | *Christensenellaceae_R-7_group* | 4.01 | 9.48 | 1.2102 | 0.0201 |
|  | *Butyrivibrio_2* | 1.51 | 1.06 | 0.3275 | 0.4994 |
|  | *Erysipelotrichaceae_UCG-004* | 1.94 | 1.41 | 0.3935 | 0.5148 |
|  | *Ruminococcaceae_NK4A214_group* | 2.88 | 5.73 | 0.8214 | 0.0823 |
|  | *Lachnospiraceae_XPB1014_group* | 1.12 | 1.47 | 0.2652 | 0.5184 |
|  | *Ruminococcus_1* | 0.87 | 0.47 | 0.1001 | 0.0438 |
|  | *Ruminococcaceae_UCG-010* | 1.50 | 1.06 | 0.1154 | 0.0586 |
|  | *Ruminococcus_2* | 0.73 | 0.99 | 0.1735 | 0.4661 |
|  | *Saccharofermentans* | 1.77 | 0.95 | 0.1882 | 0.0246 |
|  | *Ruminococcaceae_UCG-014* | 3.17 | 1.30 | 0.4693 | 0.0433 |
| Fibrobacteres | *Fibrobacter* | 0.82 | 0.53 | 0.1443 | 0.3300 |
| Spirochaetes | *Treponema_2* | 1.62 | 0.79 | 0.2576 | 0.1092 |

SEM: standard error of the mean.

**Supplementary Table 4.** Comparison of Degree, Closness centrality, and Betweenness centrality of Tibetan sheep in the summer and winter.

| Summer | | | | Winter | | | |
| --- | --- | --- | --- | --- | --- | --- | --- |
| Id | Degree | Closness centrality | Betweenness centrality | Id | Degree | Closness centrality | Betweenness centrality |
| *Prevotella_1* | 4 | 0.23622 | 49.60843 | *Rikenellaceae_RC9_gut_group* | 5 | 0.186885 | 0 |
| *Rikenellaceae_RC9_gut_group* | 3 | 0.184049 | 0 | *Prevotella_1* | 5 | 0.229839 | 770 |
| *Christensenellaceae_R.7_group* | 10 | 0.289855 | 137.56868 | *Christensenellaceae_R.7_group* | 7 | 0.186275 | 151.801889 |
| *Ruminococcaceae_UCG.005* | 7 | 0.273973 | 143.257198 | *Parabacteroides* | 10 | 0.154472 | 11.154762 |
| *Ruminococcaceae_NK4A214_group* | 7 | 0.28169 | 182.932463 | *Helicobacter* | 10 | 0.154472 | 11.154762 |
| *Treponema_2* | 10 | 0.292683 | 145.169852 | *Clostridium_sensu_stricto_1* | 4 | 0.166667 | 43.555556 |
| *Butyrivibrio_2* | 5 | 0.244898 | 61.792836 | *Allobaculum* | 8 | 0.224409 | 300.723072 |
| *Prevotellaceae_UCG.003* | 7 | 0.239044 | 27.849383 | *Pseudomonas* | 11 | 0.192568 | 69.953899 |
| *Prevotellaceae_UCG.001* | 2 | 0 | 0 | *Blautia* | 5 | 0.220077 | 56 |
| *Lachnospiraceae_XPB1014_group* | 8 | 0.273973 | 17.607098 | *Parasutterella* | 8 | 0.215094 | 59.143985 |
| *Pseudobutyrivibrio* | 8 | 0.273973 | 17.607098 | *Lachnospiraceae_NK4A136_group* | 7 | 0.198606 | 13.477323 |
| *Prevotellaceae_YAB2003_group* | 8 | 0.273973 | 17.607098 | *Prevotellaceae_UCG.003* | 6 | 0.168639 | 4.205556 |
| *Saccharofermentans* | 5 | 0.239044 | 15.314755 | *Prevotellaceae_UCG.001* | 3 | 0.14653 | 0 |
| *Ruminococcus_1* | 7 | 0.253165 | 15.397619 | *Victivallis* | 6 | 0.168142 | 58.666667 |
| *Fibrobacter* | 6 | 0.285714 | 30.564728 | *Ruminococcaceae_UCG.014* | 6 | 0.195876 | 14.344933 |
| *X.Eubacterium._coprostanoligenes_group* | 8 | 0.247934 | 48.996617 | *Eubacterium_coprostanoligenes_group* | 4 | 0.141089 | 0 |
| *Quinella* | 9 | 0.314136 | 239.410952 | *Erysipelatoclostridium* | 10 | 0.173252 | 158.507143 |
| *Ruminococcaceae_UCG.010* | 8 | 0.298507 | 161.796787 | *Fibrobacter* | 5 | 0.168639 | 0.9 |
| *Lachnospiraceae_NK3A20_group* | 7 | 0.273973 | 69.833974 | *Erysipelotrichaceae_UCG.004* | 2 | 0 | 0 |
| *Erysipelotrichaceae_UCG.004* | 9 | 0.298507 | 129.841159 | *Ruminococcaceae_UCG.010* | 10 | 0.169643 | 79.599797 |
| *Ruminococcus_2* | 8 | 0.30303 | 218.323249 | *Escherichia.Shigella* | 9 | 0.188119 | 18.341142 |
| *Terrisporobacter* | 7 | 0.239044 | 27.849383 | *Ruminococcaceae_NK4A214_group* | 11 | 0.192568 | 69.953899 |
| *Luteimonas* | 7 | 0.239044 | 27.849383 | *Turicibacter* | 10 | 0.196552 | 353.821429 |
| *Ruminococcaceae_UCG.014* | 6 | 0.247934 | 111.859931 | *Lactobacillus* | 8 | 0.154472 | 16.67381 |
| *Papillibacter* | 8 | 0.277778 | 66.121898 | *Saccharofermentans* | 4 | 0.14653 | 0 |
| *Lachnospiraceae_AC2044_group* | 4 | 0.244898 | 12.839322 | *SP3.e08* | 2 | 0 | 0 |
| *Clostridium_sensu_stricto_1* | 3 | 0.209059 | 0 | *Treponema_2* | 5 | 0.15 | 0 |
| *Fretibacterium* | 7 | 0.310881 | 146.062866 | *Prevotella_9* | 7 | 0.171171 | 111.178571 |
| *Prevotellaceae_NK3B31_group* | 4 | 0.224719 | 59 | *Lachnospiraceae_XPB1014_group* | 8 | 0.214286 | 395.277138 |
| *X.Eubacterium._ruminantium_group* | 6 | 0.285714 | 175.931772 | *Ruminococcus_1* | 11 | 0.172205 | 17.396985 |
| *Prevotellaceae_UCG.004* | 8 | 0.306122 | 120.578146 | *Ruminococcaceae_UCG.005* | 7 | 0.191919 | 132.59127 |
| *Ruminococcaceae_UCG.002* | 6 | 0.243902 | 119.905292 | *Ruminococcaceae_UCG.013* | 3 | 0.144304 | 0 |
| *probable_genus_10* | 2 | 0 | 0 | *Enterococcus* | 7 | 0.152815 | 2.261111 |
| *Oscillospira* | 3 | 0.233463 | 0 | *Bacteroides* | 7 | 0.154054 | 10.754762 |
| *Oribacterium* | 6 | 0.25641 | 65.195596 | *Romboutsia* | 6 | 0.150794 | 8.583333 |
| *Lachnoclostridium_10* | 6 | 0.257511 | 65.874675 | *Anaeroplasma* | 7 | 0.19322 | 165.869048 |
| *Roseburia* | 7 | 0.25641 | 27.389507 | *Desulfovibrio* | 4 | 0.204301 | 0 |
| *Ruminococcaceae_UCG.004* | 7 | 0.25641 | 27.389507 | *Butyrivibrio_2* | 13 | 0.193878 | 116.502783 |
| *Veillonellaceae_UCG.001* | 7 | 0.294118 | 156.571263 | *Roseburia* | 6 | 0.241525 | 783 |
| *Selenomonas_1* | 5 | 0.228137 | 14.675 | *Papillibacter* | 7 | 0.163324 | 119.585714 |
| *Ruminococcaceae_UCG.013* | 5 | 0.273973 | 73.057631 | *Akkermansia* | 8 | 0.186885 | 11.198746 |
| *Candidatus_Saccharimonas* | 7 | 0.285714 | 97.797619 | *Ruminococcus_2* | 6 | 0.230769 | 429.374457 |
| *Ruminococcaceae_UCG.001* | 6 | 0.295567 | 117.541667 | *Ruminococcaceae_UCG.002* | 8 | 0.251101 | 889.161514 |
| *Escherichia.Shigella* | 6 | 0.263158 | 208.202316 | *Lachnospiraceae_AC2044_group* | 11 | 0.172205 | 14.931353 |
| *Anaerovorax* | 5 | 0.275229 | 41.960317 | *Anaerovorax* | 11 | 0.172205 | 14.931353 |
| *Lachnospiraceae_NK4A136_group* | 3 | 0.205479 | 0 | *Ruminiclostridium_5* | 4 | 0.202847 | 56 |
| *Methanobrevibacter* | 5 | 0.288462 | 171.44624 | *Sphaerochaeta* | 3 | 0.180952 | 0 |
| *Succiniclasticum* | 7 | 0.291262 | 47.562446 | *Acinetobacter* | 6 | 0.186275 | 30.866667 |
| *Bacteroides* | 8 | 0.280374 | 178.46829 | *Thalassospira* | 3 | 0.169139 | 0 |
| *Sphingomonas* | 7 | 0.225564 | 59 | *unidentified_Lachnospiraceae* | 6 | 0.248908 | 811 |
| *Desulfovibrio* | 9 | 0.29703 | 152.636214 | *Elusimicrobium* | 2 | 0 | 0 |
| *Sphaerochaeta* | 7 | 0.288462 | 142.003495 | *Lachnospiraceae_UCG.001* | 4 | 0.141089 | 0 |
| *Ruminococcaceae_UCG.007* | 7 | 0.291262 | 79.505352 | *Bifidobacterium* | 5 | 0.206522 | 53.406397 |
| *Alistipes* | 8 | 0.247934 | 48.996617 | *Succiniclasticum* | 5 | 0.202847 | 30 |
| *Ruminiclostridium_6* | 7 | 0.29703 | 100.896634 | *probable_genus_10* | 9 | 0.188119 | 18.341142 |
| *Family_XIII_AD3011_group* | 6 | 0.234375 | 36.716675 | *Anaerotruncus* | 4 | 0.218391 | 0 |
| *Ruminiclostridium_5* | 6 | 0.234375 | 36.716675 | *Yersinia* | 8 | 0.21673 | 706.244444 |
| *U29.B03* | 6 | 0.285714 | 252.820833 | *Candidatus_Saccharimonas* | 7 | 0.207273 | 182.660966 |
| *Brevundimonas* | 9 | 0.269058 | 236.062618 | *Alloprevotella* | 8 | 0.172205 | 72.011111 |
| *Lachnospiraceae_ND3007_group* | 8 | 0.283019 | 103.14675 | *Eubacterium_ruminantium_group* | 5 | 0.163793 | 0 |
| *Anaeroplasma* | 5 | 0.26087 | 0 | *Lachnospiraceae_NK3A20_group* | 5 | 0.203571 | 10.891514 |
| *X.Ruminococcus._gauvreauii_group* | 7 | 0.265487 | 55.888092 |  |  |  |  |
| *Defluviitaleaceae_UCG.011* | 3 | 0.184615 | 0 |  |  |  |  |


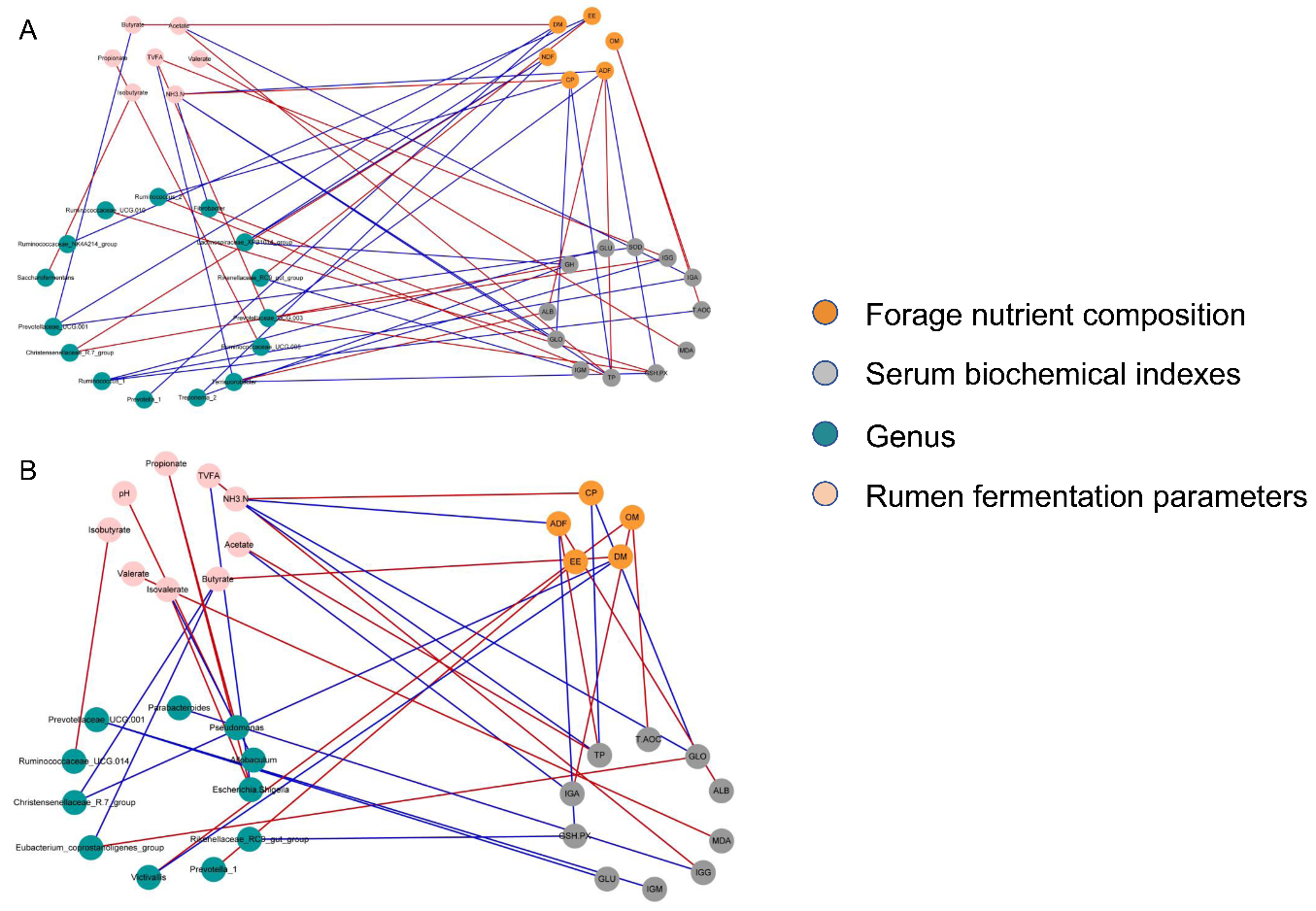


**Figure S1.** The correlation network plots among the dominant rumen bacterial genera, forage nutrient composition, rumen fermentation parameters, and serum biochemical indexes (absolute value of correlation coefficients > 0.6). The dominant rumen bacterial genera, forage nutrient composition, rumen fermentation parameters, and serum biochemical indexes are presented as dots. The red edge indicates positive correlation and blue edge indicates negative correlation. A: summer; B: winter.


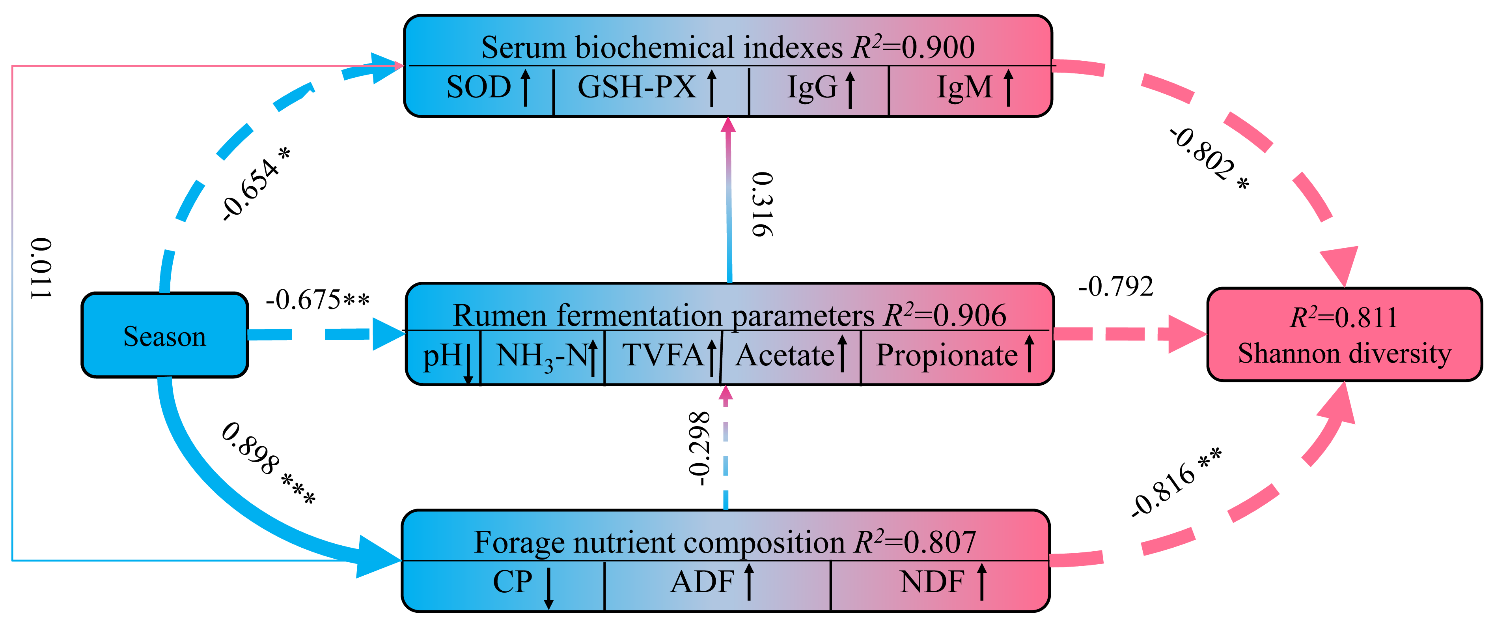


**Figure 2.** Structural equation model of the relationship between the season and forage nutrient composition, rumen fermentation parameters, serum biochemical indexes, and microbial community diversity. The relative thickness of each arrow represents the strength of the relationship (solid lines - positive relationship; dotted lines - negative relationship). ****p*<0.001; ***p*<0.01; **p*<0.05. Results of model fitting: χ^2^ =0.45, *df* = 1, *P* = 0.51.
